# Supplementary material for: MetaRibo-Seq measures translation in microbiomes
Source: Nat Commun. 2020 Jun 29;11:3268. doi: 10.1038/s41467-020-17081-z (PMC7324362; doi:10.1038/s41467-020-17081-z)
Supplement: Supplementary file 10 — Supplementary Data 7 [file 41467_2020_17081_MOESM10_ESM.zip › File2/Confidence_High_Taxonomy/277588_out.krona.html]

Javascript must be enabled to view this page.

members
magnitude
magnitudeUnassigned
count
unassigned
taxon
rank

277588\_out

26

2
superkingdom
26

1239
phylum
26

26
class
186801

186802
order
26

SRS014412\_contig\_number\_contig-100\_23204.23205SRS016056\_contig\_number\_contig-100\_17836.17836SRS018817\_contig\_number\_contig-100\_7369.82879SRS047433\_contig\_number\_contig-100\_3745.3746SRS049773\_contig\_number\_contig-100\_34873.34874SRS050925\_contig\_number\_contig-100\_4461.4461
6

family
7
31979

7
genus
1485

7
species

SRS014613\_contig\_number\_1401SRS018313\_contig\_number\_contig-100\_18.62777SRS020869\_contig\_number\_11084SRS045645\_contig\_number\_17193SRS098644\_contig\_number\_33558SRS1054691\_contig\_number\_17785SRS143598\_contig\_number\_4555
59620

family
13
186803

841
1
genus


SRS148874\_contig\_number\_8000
512314
species
1

1898203

SRS014459\_contig\_number\_contig-100\_2470.214833SRS015065\_contig\_number\_24457SRS015190\_contig\_number\_contig-100\_4268.4268SRS015854\_contig\_number\_contig-100\_3634.116622SRS024435\_contig\_number\_24621SRS045004\_contig\_number\_28419SRS048060\_contig\_number\_6088SRS077194\_contig\_number\_9165SRS098881\_contig\_number\_contig-100\_2384.166225SRS143417\_contig\_number\_24140SRS144537\_contig\_number\_32970SRS148159\_contig\_number\_54572
12
species
